# Employee political skill, leader-member exchange, and promotability: the moderating role of task performance

**DOI:** 10.3389/fpsyg.2026.1855375

**Published:** 2026-06-01

**Authors:** Congyong Shang, Songlin Chen, Zhidong Li

**Affiliations:** 1 College of Economics and Management, Talent Development Institute, Hefei Normal University, Hefei, China; 2 School of Public Administration, Anhui Jianzhu University, Hefei, China

**Keywords:** LMX, political skill, promotability, social exchange theory, task performance

## Abstract

Extant research has identified that employee political skill exerts a positive influence on the formation of high-quality leader-member exchange (LMX), thereby impacting career outcomes. However, the boundary conditions that moderate this relationship, especially from an integrated career capital and social exchange perspective, remain underexplored. Grounded in these two theoretical perspectives, we argue that task performance, as human capital, enables political skill, conceptualized as social capability or relational competence, to translate into high-quality LMX as realized relational social capital. Specifically, we propose that task performance strengthens the political skill–LMX link, which in turn enhances promotability. Analyses of 200 supervisor-subordinate dyads support our hypotheses. These results yield substantive implications for organizational behavior theory and managerial practice.

## 1 Introduction

Organizations function as natural political arenas (Mintzberg, 1985). To thrive within such environments, employees must possess the aptitude to assess situations, forge alliances, and influence others-aptitudes that Ferris et al. (2007) collectively define as political skill. Previous studies have found that employee political skill contributes to LMX, and LMX serves as a vital psychological mechanism that connects employee political skill to employee career outcomes (Epitropaki et al., 2016; Magnusen and Kim, 2016). Against this backdrop, the relationship between political skill and career advancement has garnered increasing scholarly attention, with notable indigenous research emerging in the Chinese context. Tang et al. (2019) documented that political skill influences promotability through supervisor-subordinate guanxi (SSG), with team power distance moderating this process. This work establishes the empirical relevance of political skill for career outcomes in Chinese organizations.

Despite these insights, two important research gaps remain unaddressed. First, while Tang et al. (2019) focused on contextual-cultural conditions (team power distance) that enable leaders to favor certain subordinates, it remains unclear under what individual conditions leaders choose to invest in subordinates. Second, their use of the indigenous construct of guanxi, while valuable for capturing culture-specific dynamics, limits dialogue with the broader international literature that predominantly employs LMX as the theoretical lens for supervisor-subordinate relationships.

Recent international research has also advanced this line of inquiry but still leaves critical questions unanswered. Studies have explored how environmental factors such as organizational politics moderate the political skill-LMX relationship (Li et al., 2025), focusing on employees’ motivation to build relationships in response to environmental uncertainty. Yet, this work leaves unexamined the leader-driven side of the equation: under what conditions do leaders actually accept and reciprocate employees’ relational overtures? Furthermore, while recent research has documented both bright and dark outcomes of political skill (Cui and Zhang, 2025; Zahid et al., 2025), but few have considered how task performance legitimizes such relational efforts.

To address these gaps, we integrate career capital theory (DeFillippi and Arthur, 1994; Arthur et al., 1995) and social exchange theory (Blau, 1964) to examine how task performance, as human capital, enables political skill, viewed as a form of social competence or ‘relational capability’, to translate into career success via LMX as realized relational social capital. Career capital theory conceptualizes human capital and relational capability as complementary foundations for career advancement.

In line with this integrated framework, we argue that the positive effect of employee political skill cannot be fully understood without considering the critical boundary role of task performance. From the perspective of career capital theory (DeFillippi and Arthur, 1994), political skill represents a vital relational capability that does not automatically translate into actual relational resources; its effectiveness depends on whether it is backed by demonstrable competence. Task performance, defined as behaviors that fulfill core job requirements and enhance the organizational technical core (Borman and Motowidlo, 1993), is the most relied upon workplace capital for employees in an organization (Wang and Duan, 2015). Within the framework of social exchange theory (Blau, 1964), supervisor-employee interaction essentially constitutes a process of resource exchange where supervisors tend to allocate limited resources (e.g., time, support) to high-value employees to maximize returns. Even when politically skilled employees initiate interactions (Ferris et al., 2005a), whether supervisors respond positively depends on their rational judgement of the employee’s value.

Building on this logic, we propose a more nuanced interpretation: social capability (political skill) can only be effectively converted into actual relational resources (high-quality LMX) when supported by sufficient human capital (task performance). Human capital thus serves as a legitimacy endorsement that enables relational capability to bear fruit. Employees with high task performance, recognized as “high-value partners” by their supervisors due to their high contribution to the organization’s key goals (Borman and Motowidlo, 1993), are more likely to gain positive responses in their interactions. Thus, the association between employee political skill and LMX can be stronger under high task performance. We further posit that this combined effect will shape promotability via LMX as a mediator.

**Figure 1:**
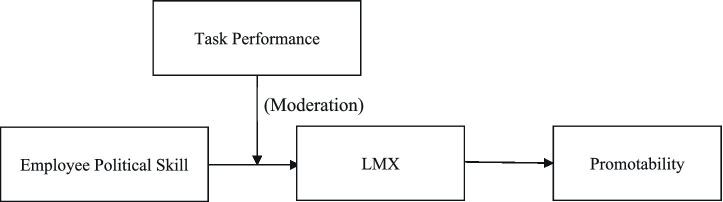
Theoretical model. LMX, leader-member exchange.

Overall, this research yields three principal contributions. First, we integrate career capital theory with social exchange theory to advance LMX research, revealing that LMX formation involves a dynamic process of capital conversion: relational capability (political skill) transforms into relational social capital (LMX) when legitimized by human capital (task performance). Second, we identify task performance as a critical enabling condition that determines when political skill translates into LMX and subsequent promotability. Third, by situating our findings alongside Tang et al.’s (2019) cultural contingency and Li et al.’s (2025) environmental contingency, we provide a tripartite framework for understanding how political skill operates across cultural, environmental, and individual levels. Figure 1 illustrates the theoretical model of this study.

## 2 Theory and hypotheses

### 2.1 The moderating role of task performance

Building on career capital theory (DeFillippi and Arthur, 1994; Arthur et al., 1995), we conceptualize political skill as social capability or relational competence that facilitates interpersonal influence and relationship development, while task performance represents essential human capital signaling employee value. Career capital theory holds that relational capability alone cannot drive career gains; it must be enabled and legitimized by high-quality human capital to be considered worthy of organizational investment. Integrating social exchange theory (Blau, 1964), we propose that task performance strengthens the translation of political skill into high-quality LMX as realized relational social capital.

Social exchange theory (Blau, 1964) posits that social exchange entails reciprocal interactions: individuals reciprocate positive treatment, and through repeated engagement, both parties may develop high-quality exchange relationships marked by mutual trust and loyalty (Cropanzano and Mitchell, 2005). While prior LMX research has focused on this reciprocity principle, less attention has been paid to the rationality principle (Meeker, 1971). This principle holds that willingness to engage also depends on two forward-looking judgments: first, whether others’ positive treatment is sincere, and second, whether one’s own investment will yield future returns rather than disappointment (Cropanzano and Mitchell, 2005; Meeker, 1971).

Political skill is a multidimensional construct encompassing social astuteness, interpersonal influence, apparent sincerity, and networking ability. It reflects one’s ability to comprehend social dynamics and shape others’ perceptions and behaviors (Ferris et al., 2005a; Ferris et al., 2007). Specifically, social astuteness enables accurate interpretation of others’ behaviors; interpersonal influence allows flexible adaptation to situational demands; apparent sincerity projects genuine concern and fosters trust; and networking ability facilitates building and leveraging connections. These competencies collectively enable politically skilled employees to understand supervisor expectations, adapt their behavior accordingly, interact sincerely, and forge strong relational bonds (Ferris et al., 2007; Wihler et al., 2017). Recent studies supports the positive link between political skill and LMX (Cui and Zhang, 2025; Zahid et al., 2025), though it may also yield dysfunctional outcomes.

Drawing on career capital and social exchange theories, we argue that the political skill-LMX relationship hinges on task performance. The transformation of political skill into high-quality LMX unfolds through a three-stage, supervisor-mediated process. In the capability display stage, politically skilled employees initiate relational overtures, which constitute only potential social capital. In the value screening stage, supervisors apply the rationality principle (Meeker, 1971), using task performance as a legitimacy test: high performance validates the employee’s worth and signals future returns, thereby legitimizing relational efforts. In the resource commitment stage, supervisors translate approved potential into tangible support, forming high-quality LMX. Thus, task performance acts as the critical screening gate that determines whether relational capability is converted into relational social capital.

From a career capital perspective, high task performance (as robust human capital) legitimizes relational efforts by demonstrating substantive contribution. From a social exchange rationality perspective, it satisfies the supervisor’s forward-looking calculus, assuring that investment in relational overtures will be reciprocated with continued value creation. The convergence of these lenses reveals a process of capital conversion: high-performing employees possess both the legitimacy and the perceived future value needed to convert political skill into high-quality LMX. Relational overtures are thus framed not as one-way demands but as the active fulfillment of reciprocal obligations grounded in substantive competence.

Conversely, low task performance disrupts the conversion at the value-screening stage. Career capital theory suggests that without the foundation of human capital, relational capability appears suspect, reflecting a “resource-compensation strategy” to offset performance deficiencies through relational means. Social exchange theory further explains the fallout. From the reciprocity perspective, the imbalance may evoke perceptions of exploitation (Gouldner, 1960), leading supervisors to dismiss such behaviors as “instrumental tactics” or “political maneuvering” to conceal poor productivity. From the rationality perspective, supervisors exercise cost–benefit caution, recognizing that investing in low-performing employees, however politically skilled, yields poor returns. Consequently, supervisors limit interaction and withhold resources, attenuating the association between political skill and LMX. Therefore, we propose:

*Hypothesis 1*: Employee task performance moderates the relationship between political skill and LMX, such that this relationship is stronger when employee task performance is high (versus low).

### 2.2 Moderated mediation effect on Promotability

We further argue that the interplay between employee political skill and task performance shapes promotability through LMX. LMX theory posits that supervisors develop exchange relationships of varying quality with subordinates (Graen and Uhl-Bien, 1995). LMX, conceptualized as realized relational social capital, has been robustly linked to promotability (Rockstuhl et al., 2012; Schaubroeck and Lam, 2002). High-quality LMX provides deeper socio-emotional bonds (trust, liking, respect, and support) (Erdogan and Bauer, 2014) and instrumental benefits (access to supervisors’ private networks and career development resources), thereby facilitating promotions (Seibert et al., 2001; Han, 2010).

Career capital theory posits that relational social capital yields the greatest returns when its acquisition is legitimized by robust human capital (DeFillippi and Arthur, 1994; Arthur et al., 1995). This conditional logic directly applies to the translation of political skill into LMX: the conversion of relational capability into relational social capital depends on task performance as the legitimizing human capital.

Integrating career capital theory with social exchange theory’s reciprocity and rationality principles, we propose that task performance moderates the mediating role of LMX in the political skill-promotability relationship. When task performance is high, supervisors perceive politically skilled employees’ relational initiatives as credible signals grounded in demonstrated competence, anticipating future value (rationality principle). High task performance legitimizes political skill, enabling its conversion into high-quality LMX and, subsequently, higher promotability. When task performance is low, political skill is discounted as manipulative or compensatory; concerns over exploitation and poor future returns weaken the political skill–LMX link, diminishing the indirect path to promotability. Therefore, we hypothesize:

*Hypothesis 2*: Employee task performance moderates the mediation effect of political skill on promotability via LMX, such that this mediation effect is stronger when employee task performance is high (versus low).

## 3 Methods

### 3.1 Samples and procedure

To avoid common method bias (CMB), we employed supervisor-employee paired data for empirical analysis. Because obtaining a sufficient quantity of valid paired data proved challenging, we adopted a methodology utilized in prior research: enlisting intermediaries to conduct online surveys (Greenbaum et al., 2018). Intermediaries were recruited through academic and professional networks using three criteria: (1) being full-time employees with stable organizational roles; (2) having legitimate, professional working connections with potential participants; and (3) commitment to research ethics and ensuring anonymous, honest survey implementation. Their relationships with participants were neutral: intermediaries served solely as facilitators for questionnaire distribution and code matching, and held no hierarchical authority over, nor any conflicting personal ties with, any of the participating employees or supervisors.

Before conducting our official survey, we detailed the survey procedure to each intermediary. (1) Randomly select up to five employees within each sample organization. (2) Distribute the “Employee Survey” link to the selected employees and instruct them to complete the “Employee Behavior” survey. Upon completion, they should send the completed “code” to the intermediary. (3) The intermediary then forwards this “code” (required by supervisors when filling out their surveys) along with the corresponding employee’s name and the “supervisor Survey” link to the employee’s direct supervisor for completion. (4) After receiving the “code,” we verify each completed survey within 48 h. We rewarded each validated paired survey with a payment of 10 yuan. We took special care to ensure that if either the employee or supervisor survey was suspicious, the entire paired surveys were deemed invalid. Additionally, we highlighted three key points to the intermediaries. (1) The survey was anonymous and intended solely for academic research; maintaining integrity is paramount. (2) Both employees and supervisors were asked to enter the same “code” in their surveys to ensure valid pairing. (3) Supervisors were reminded to accurately provide their commonly used email addresses, as this is a critical factor for verifying the validity of the survey and facilitating the tracking and random verification of the sample source. As a result, a total of 209 questionnaires were collected, comprising responses from 149 supervisors and 209 employees. After excluding mismatched supervisor-employee dyadic questionnaires, 200 valid matched pairs were ultimately retained. This final dataset included 148 supervisors (with a valid response rate of 99.33%) and their corresponding 200 employees (showing a valid response rate of 95.69%). Each supervisor assessed between 1 and 4 employees, with an average of 1.35 employees.

The participants represented a variety of organizational types: private enterprises (39.5%), state-owned enterprises (22.5%), public institutions (20.5%), government departments (7.0%), foreign-owned companies (2.0%), joint ventures (0.5%), and “other” (8.0%). The demographic breakdown of the employees included 55.5% male and 44.5% female; age distribution was as follows: 21.5% were aged 25 or younger, 42.5% were between 26 and 30, 25.0% were between 31 and 35, 8.0% were between 36 and 40, 2.0% were between 41 and 45, 0.5% were between 46 and 50, and 0.5% were over 51 years old. Educational level showed that 20.0% had an associate degree or below, 62.5% possessed a bachelor’s degree, 16.5% held a master’s degree, and 1.0% held a doctorate. Job tenure (employee’s duration in a particular position) was as follows: 16.0% lasting up to 1 year, 28.5% spanning between 1 and 3 years, 23.0% enduring for 4 to 6 years, and 32.5% exceeding 7 years. Organizational tenure (employee’s duration at the current organization) was as follows: 33.0% lasting up to 1 year, 33.5% spanning between 1 and 3 years, 17.0% enduring for 4 to 6 years, and 16.5% exceeding 7 years.

Among the supervisors, the gender breakdown included 59.5% males and 40.5% females. The age distribution included 3.5% aged 25 or below, 27.0% between 26 and 30, 38.0% between 31 and 35, 20.0% between 36 and 40, 7.5% between 41 and 45, 3.0% between 46 and 50, and 1.0% over 51. The educational level among supervisors was 15.0% with an associate degree or below, 57.0% possessing a bachelor’s degree, 27.0% possessing a master’s degree, and 1.0% possessing a doctorate. Supervisor-employee tenure (the duration of supervisor-employee relationships) was as follows: 29.5% lasting up to 1 year, 37.5% spanning between 1 and 3 years, 20.0% enduring for 4 to 6 years, and 13.0% exceeding 7 years.

### 3.2 Measures

Given that our measurement instruments were originally in English, we enlisted two management Ph.D. scholars, proficient in English as well as Chinese, to adapt our English versions for Chinese, utilizing the back-translation process proposed by Brislin (1970). For all key variables, a five-point Likert scale was adopted, wherein 1 signified “strongly disagree” and 5 signified “strongly agree”.

#### 3.2.1 Employee political skill scale

The18-item instrument constructed by Ferris et al. (2005b) was adopted to assess employee political skill (Cronbach’s *α* = 0.94). A representative item is, “I understand people very well”.

#### 3.2.2 Task performance scale

Supervisors rated employee task performance with the five-item instrument utilized by Janssen and Van Yperen (2004) (Cronbach’s *α* = 0.78). An example item is, “This employee fulfills all responsibilities required by his/her job”.

#### 3.2.3 LMX scale

Supervisor rated LMX adopting the 5-item instrument utilized by Raghuram et al. (2017) (Cronbach’s *α* = 0.83). A representative item is, “I feel that my employee understands my problems and needs”.

#### 3.2.4 Promotability scale

Supervisor rated promotability via the 3-item measure utilized by Hoobler et al. (2009) (Cronbach’s *α* = 0.84). An example item is, “I believe that this employee has high potential”.

#### 3.2.5 Control variables

In this study, employee gender, age, education level, supervisor-employee tenure, job tenure, and organizational tenure were treated as controls due to their potential to impact LMX and promotability (Thacker and Wayne, 1995; Wayne et al., 2002).

### 3.3 Analytic strategy

First, confirmatory factor analysis (CFA) was applied to test the discriminant validity of the study’s key variables. Second, reliability tests (i.e., Cronbach’s *α*) and descriptive statistics were conducted using SPSS 22. Finally, our hypotheses were analyzed via Mplus 8. Our variables were all individual-level variables; however, to account for data nesting (supervisors rated 1–4 employees each), the “cluster” and “type = complex” commands in Mplus were applied to control nested effects. This method utilizes the “sandwich estimator” to ensure robust standard errors under non-independent sampling, thereby controlling for nesting effects (Muthén and Muthén, 2007).

Moreover, we conducted supplementary analysis: two-stage moderation test. To exclude the alternative possibility that task performance moderates the linkage between LMX and promotability in the second stage, we performed a simultaneous two-stage moderation analysis via the SPSS PROCESS macro (Model 59; Hayes, 2013). This framework simultaneously assesses whether task performance moderates the association between political skill and LMX in the first stage, as well as the relationship between LMX and promotability in the second stage. It also accounts for the moderating influence on the residual direct association from political skill to promotability within one unified analytical setting.

## 4 Results

### 4.1 Preliminary analyses

#### 4.1.1 Correlations

Descriptive statistics and correlations are presented in Table 1.

**Table 1:** Descriptive statistics and correlations.

Variable	*M*	SD	1	2	3	4	5	6	7	8	9	10
1. Gender	1.45	0.50										
2. Age	2.30	1.04	−0.17^*^									
3. Degree	1.99	0.64	0.16^*^	0.12								
4. Supervisor-employee tenure	2.17	1.00	−0.11	0.31^**^	−0.19^**^							
5. Job tenure	2.72	1.09	−0.11	0.64^**^	−0.12	0.48^**^						
6. Organizational tenure	2.17	1.07	−0.08	0.48^**^	−0.14	0.65^**^	0.64^**^					
7. Employee political skill	3.94	0.66	−0.29^**^	0.00	−0.18^*^	0.15^*^	0.03	0.08	0.94			
8. Task performance	4.30	0.60	−0.06	0.07	0.07	−0.05	0.09	0.03	0.03	0.78		
9. LMX	4.22	0.62	0.11	0.03	0.19^**^	−0.04	0.09	0.00	0.07	0.60^**^	0.83	
10. Promotability	4.23	0.76	0.02	−0.02	0.23^**^	−0.15^*^	−0.04	−0.12	0.10	0.69^**^	0.67^**^	0.84

*N* = 200. LMX, leader-member exchange. Cronbach’s α coefficients are presented on the diagonal in parentheses.

^*^p < 0.05, ^**^p < 0.01.

#### 4.1.2 Confirmatory factor analysis (CFA)

Before proceeding with hypotheses testing, CFAs were carried out to examine the discriminant validity of the primary constructs: employee political skill, task performance, LMX, and promotability. This was done to ascertain that each construct measured what it was intended to measure without significant overlap with the others.

According to Epitropaki et al. (2016), we packaged the political skill construct consisting of four factors: taking the mean of the items for each of the four factors as the item for the political skill measure. Our proposed measurement model demonstrated excellent fit, with *χ*^2^ = 161.90, *df* = 113, *p* < 0.001, RMSEA = 0.05, CFI = 0.97, and SRMR = 0.05. Notably, this model significantly outperformed each of the potential alternative models, including three three-factor models (Δ*χ*^2^ (3) ranging from 66.31 to 602.72, all *p* < 0.001), a two-factor model (Δ*χ*^2^ (5) = 1062.89, *p* < 0.001), and a one-factor model (Δ*χ*^2^ (6) = 1329.49, *p* < 0.001).

#### 4.1.3 CMB test

Given that task performance, LMX, and promotability were all rated by supervisors, we conducted supplementary CFAs. Findings indicated that the three-factor model (*χ*^2^ = 95.36, *df* = 62, *p* < 0.001, RMSEA = 0.05, CFI = 0.98, SRMR = 0.04) provided a superior fit compared with each of the two-factor models (Δ*χ*^2^(2) = 65.93, Δ*χ*^2^(2) = 94.14, both *p* < 0.001) and a one-factor model (Δ*χ*^2^(3) = 124.51, *p* < 0.001). Furthermore, Harman’s single-factor test showed that the first factor explained 29.94% of the total variance, which is below the 40% threshold commonly used to indicate CMB. As noted by Podsakoff et al. (2003), these results indicated that our primary measures possessed ample discriminant validity, while also suggesting that CMB did not pose a serious issue.

### 4.2 Hypotheses tests

**Figure 2:**
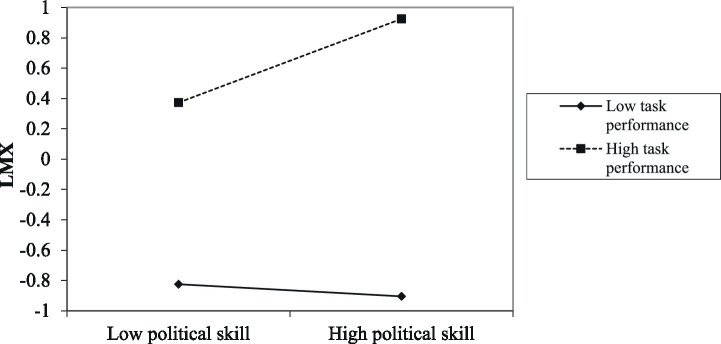
Interaction of employee political skill and task performance on LMX. The dashed line represents high task performance (M + 1 SD), and the solid line represents low task performance (M-1 SD). LMX, leader-member exchange.

Results for Hypothesis 1 are presented in Table 2. The interaction of employee political skill and task performance significantly influenced LMX (*β* = 0.16, *SE* = 0.07, *p* < 0.05; see Table 2), supporting Hypothesis 1. Simple slope tests showed that at low level of task performance, the relationship between employee political skill and LMX was not significant (*β* = −0.04, *SE* = 0.11, *p* = 0.72 > 0.05), but at high level of task performance, it was significantly positive (*β* = 0.28, *SE* = 0.09, *p* < 0.01; see Figure 2).

**Table 2:** Coefficients of the model for testing task performance moderation.

Variable	LMX	
	*β*	SE
Gender	0.16^**^	0.06
Age	−0.07	0.08
Degree	0.17^**^	0.06
Supervisor-employee tenure	−0.05	0.09
Job tenure	0.15	0.09
Organizational tenure	−0.01	0.09
Employee political skill	0.12	0.07
Task performance	0.76^***^	0.07
Employee political skill × task performance	0.16^*^	0.07
Intercept	−0.11	0.07
Residual	0.56^***^	0.06

*N* = 200. Standardized coefficients are reported. LMX, leader-member exchange.

^*^p < 0.05; ^**^p < 0.01; ^***^p < 0.001, two-tailed tests.

For Hypothesis 2, our results provided empirical support. Specifically, when task performance was low, the conditional indirect effect of employee political skill on promotability through LMX amounted to −0.02 (95%CI = [−0.12, 0.08], non-significant). In contrast, when task performance was high, this effect reached 0.16 (95%CI = [0.08, 0.25]). The between-condition difference amounted to 0.18 (95%CI = [0.05, 0.32]), indicating that employee political skill exerted a statistically more pronounced indirect effect on promotability through LMX in high-task-performance contexts than in low-task-performance scenarios. Thus, Hypothesis 2 was supported.

Additionnaly, we conducted supplementary analysis: ruling out second-stage moderation. Using PROCESS Model 59 (Hayes, 2013), we simultaneously tested the moderating effects of task performance on both the first stage (political skill → LMX) and the second stage (LMX → promotability), as well as the residual direct effect (political skill → promotability). The results confirmed our theoretical prediction: the first-stage moderation was significant (*β* = 0.12, SE = 0.06, *p* = 0.048, 95% CI [0.001, 0.25]), replicating our primary findings. By contrast, the second-stage moderation was not significant (*β* = −0.01, SE = 0.05, *p* = 0.86 > 0.05, 95% CI [−0.11, 0.09]). The residual direct effect moderation was also not significant (*β* = 0.02, *p* = 0.66 > 0.05). Furthermore, the conditional indirect effects replicated the moderated mediation pattern: non-significant at low task performance (*β* = 0.01, 95% CI [−0.04, 0.06]), but significant at high task performance (*β* = 0.08, 95% CI [0.03, 0.16]). Collectively, these results rule out the competing explanation and strengthen the conclusion that task performance’s moderating role is limited exclusively to the first-stage capital conversion process.

## 5 Discussion

Our analysis of data from 200 supervisor-employee dyads across various organizational types found that: (1) task performance exhibited a significant moderating effect on the association between employee political skill and LMX. Specifically, at low levels of task performance, this relationship was not significant, but significant at high levels; and (2) task performance positively moderated the indirect pathway from employee political skill to promotability through LMX. Specifically, at low levels of task performance, this indirect effect was not significant, but significant at high levels.

### 5.1 Theoretical implications

This study contributes four primary theoretical insights. First, it reveals the conversion mechanism between human capital and relational capability, advancing the application of career capital theory by moving from a static resource portfolio view to a dynamic capital conversion perspective. Career capital theory emphasizes the synergistic influence of multiple capitals on career success (Fugate et al., 2004), but there has been a lack of empirical testing on how they synergize. This study finds that task performance (human capital) is the boundary condition for political skills (relational capability) to be converted into actual relational resources—only when task performance is high can political skills be effectively transformed into LMX as realized social capital and subsequently enhance promotion opportunities. This finding reveals the endorsement effect of human capital on relational capability, deepening the synergy proposition of career capital theory into a hierarchical conversion relationship: human capital legitimizes relational capability, enabling it to be cashed out as tangible relational resources.

Second, it revives the rational principle of social exchange theory and expands the understanding of the formation mechanism of LMX. Existing LMX research has mainly focused on the reciprocity principle, neglecting the role of the rational principle (Cropanzano and Mitchell, 2005). We find that leaders do not blindly respond to employees’ social overtures but make rational investment decisions-only high-performing employees are worth establishing high-quality LMX. This rational logic is consistent with the broader meta-analytic finding that individual competencies matter for LMX (Dulebohn et al., 2012). Also, the cross-cultural robustness of LMX documented by Rockstuhl et al. (2012) suggests that this rational logic may generalize to the Chinese organizational setting. This finding shifts the leadership position from a passive reciprocity to an active investor, providing a new theoretical perspective for understanding leader-member relationships.

Third, by complementing local research, the understanding of promotability mechanisms in the Chinese organizational context has been deepened. Tang et al. (2019) found that team power distance (whether leaders can be partial) moderates the relationship between SSG and promotability. This study, on the other hand, reveals that task performance (whether leaders are willing to invest) moderates the relationship between political skills and LMX. Both studies jointly demonstrate that in the Chinese organizational context, obtaining promotability requires both cultural conditions (power distance) of “leaders being able to be partial” and personal conditions (task performance) of “leaders being willing to invest”. This challenges the stereotype of “relationships being everything”, indicating that in Chinese organizations, ability remains the foundation for relationships to play a role.

Fourth, this study engages in a dialogue with the international cutting-edge research by Li et al. (2025) as well as recent dark-side findings (Cui and Zhang, 2025; Zahid et al., 2025), constructing a more comprehensive theoretical landscape. Li et al. (2025) started from the perspective of social information processing theory and found that organizational political perception (environmental uncertainty) activates employees’ motivation for relationship building, strengthening the relationship between political skills and LMX. This study, on the other hand, adopts a perspective of rational decision-making by leaders and discovers that task performance (individual ability) determines whether leaders respond to employees’ social overtures. Combining the two reveals a two-stage process of how political skills influence career success: employees proactively make overtures based on the environment (Li et al., 2025), while leaders rationally screen based on ability (this study). This screening process is precisely the capital conversion mechanism we identify, wherein human capital determines whether relational capability yields relational resources, distinct from when it yields negative outcomes (Cui and Zhang, 2025; Zahid et al., 2025).

### 5.2 Practical implications

Our research yields important practical implications for both employees and their organizations. For employees, several key insights emerge. First, the foundational role of task performance must be emphasized. This study demonstrates that political skills are only effective when paired with strong task performance. Employees should therefore prioritize competence-focusing on enhancing their core work abilities and fulfilling job responsibilities. Only on this basis can political savvy come into play, for instance, by developing political skills through methods such as developmental simulations and behavior modeling (Ferris et al., 2002) to foster strong relationships with leaders. It is crucial to avoid misplaced priorities-excessive investment in relationship-building at the expense of performance improvement. Second, employees should develop an accurate understanding of the nature of LMX. Leaders do not simply reciprocate goodwill; rather, they rationally invest in employees who demonstrate potential. By consistently delivering high performance, employees can signal to their leaders that they are worth investing in.

For organizations, this study provides novel evidence to encourage employees to enhance their task performance. While prior research has largely focused on antecedents of task performance (Jang, 2022; Alaybek et al., 2022), our study positions task performance as a key moderating variable. This highlights its critical role in amplifying the effect of political skill on LMX and, ultimately, on promotability. Organizations can therefore draw on our theoretical framework and empirical findings to motivate employees to invest more time and energy in improving task performance-rather than relying solely on political skill to build high-quality LMX for career advancement.

### 5.3 Limitations and future directions

Our research is accompanied by four primary limitations. First, the cross-sectional design precludes causal inferences and raises the possibility of reverse causality (e.g., high-quality LMX may reversely promote employees’ political skill and task performance). Because all variables were collected simultaneously, we cannot definitively confirm the proposed causal order. Therefore, our findings should be interpreted as correlational support for the theoretical model, not as evidence of causal direction. We recommend that future research employ a multi-wave longitudinal panel design, for example, a three-wave time-lagged design (Wave 1: political skill and task performance; Wave 2: LMX; Wave 3: promotability), and use cross-lagged panel models or random intercept cross-lagged panel models to rigorously test reciprocal effects and strengthen causal inference (Ployhart and Vandenberg, 2010). Future research could further employ longitudinal experimental designs (e.g., training interventions to improve political skill or task performance). This would provide even stronger causal evidence for the proposed model.

Second, our model omits several other key variables that may influence the core relationships or serve as alternative mediating pathways. OCB may strengthen the political skill-LMX link (Organ, 1988) and, because it shapes supervisory evaluations via affective mechanisms (Allen and Rush, 1998), could similarly moderate this relationship. Recent evidence confirms political skill positively relates to OCB, with attachment style moderating this link (Bruny et al., 2023). Personality traits may confound effects (Ferris et al., 2007); meta-analytic evidence identifies extraversion and conscientiousness as antecedents of political behavior (Frieder et al., 2024). Workload could constrain supervisors’ capacity to engage in high-quality exchanges, and job level may determine the relative weight of political skill versus task performance in promotability decisions. Industry characteristics matter, as political skill dimensions vary across industries (Tiwari et al., 2025). Moreover, LMX alone may not capture the full “knowing-whom” dimension; political skill may also build peer and external networks. Indeed, political skill facilitates networking behaviors that shape outcomes (Wanigasekara et al., 2026). Future research could incorporate these variables into the model and adopt a social network perspective to examine how political skill functions through multiple relational pathways to build realized social capital and facilitate promotability.

Third, we presents several measurement limitations. (1) Residual CMB may remain. We adopted a multi-source design: employees self-rated their political skill, and supervisors assessed task performance, LMX and promotability. Both CFA and Harman’s single-factor test confirmed that CMB exerted only a minor influence on the data. Nevertheless, three of the four core variables were rated by the same supervisor, which may introduce residual common variance. (2)Self-reported political skill is vulnerable to social desirability bias. Since apparent sincerity is a core dimension of political skill (Ferris et al., 2005a), respondents may strategically provide favorable self-ratings. While anonymity can mitigate this concern (Podsakoff et al., 2003), exclusive reliance on self-reports without supervisor or peer evaluations constitutes a methodological limitation. Notably, self-report measurement remains the mainstream and well-validated approach in political skill research (Ferris et al., 2005a; Epitropaki et al., 2016), and recent studies still adopt this method to explore its antecedents and outcomes (Cui and Zhang, 2025; Li et al., 2025; Zahid et al., 2025; Rao et al., 2026). To enhance measurement validity, future research could adopt multi-source ratings from supervisors or peers, statistically control for social desirability, triangulate data via additional evaluators, or implement full cross-source and multi-wave designs.

Fourth, the sample representativeness is limited, and the sampling method carries a risk of bias. The age distribution is skewed (67.5% aged 26–35; only 0.5% over 51), and organizational types are unbalanced (foreign-funded and joint ventures = 2.5%), restricting the generalizability of our findings to these contexts. Moreover, this study adopted online snowball sampling through intermediaries, which may introduce selection bias and reduce sample randomness, thereby limiting the generalizability of the results. Thus, our findings should be interpreted with caution when extending to older employee populations or to underrepresented organizational types. Future research could adopt more balanced sampling strategies, such as stratified random sampling or quota sampling to improve representativeness. Cross-validation of our findings in diverse samples (e.g., a broader age range and more foreign-owned enterprises) would further strengthen the generalizability of the conclusions.

## 6 Conclusion

Based on social exchange theory and the political skill literature, this study offers significant insights regarding how employee political skill influences promotability with task performance as a moderator. Conceptualizing political skill as relational capability and LMX as realized relational social capital, we find that employee task performance enhances the conversion of political skill into LMX. Moreover, employee political skill has a stronger indirect influence on promotability via LMX under high (vs. low) task performance. In other words, employees with high task performance exert stronger influence on their interaction with supervisors by leveraging their political skill, strengthening LMX quality and, in turn, promotability. These insights underscore the hierarchical synergy between human capital and relational capability in driving career advancement: competence first enables connections to flourish..

## Data Availability

The raw data supporting the conclusions of this article will be made available by the authors, without undue reservation.

## References

[ref1] Alaybek B. Wang Y. Dalal R. S. Dubrow S. Boemerman L. S. G. (2022). The relations of reflective and intuitive thinking styles with task performance: a meta-analysis. Pers. Psychol. 75, 295–319. doi: 10.1111/peps.12443

[ref2] Allen T. D. Rush M. C. (1998). The effects of organizational citizenship behavior on performance judgments: a field study and a laboratory experiment. J. Appl. Psychol. 83, 247–260. doi: 10.1037/0021-9010.83.2.247, 9577234

[ref3] Arthur M. B. Claman P. H. DeFillippi R. J. (1995). Intelligent enterprise, intelligent career. Acad. Manage. Exec. 9, 7–20. doi: 10.5465/ame.1995.9512032185

[ref4] Blau P. M. (1964). Exchange and power in social life. Hoboken, NJ: Wiley.

[ref5] Borman W. C. Motowidlo S. J. (1993). “Expanding the criterion domain to include elements of contextual performance,” in Human Performance, eds. Schmitt N. Borman W. C. (San Francisco, CA: Jossey-Bass).

[ref6] Brislin R. W. (1970). Back-translation for cross-cultural research. J. Cross-Cult. Psychol. 1, 185–216. doi: 10.1177/135910457000100301

[ref7] Bruny J. F. Vallée B. Bernardi F. Rioux L. Scrima F. (2023). Workplace attachment style as moderator of the relationship between political skills and organizational citizenship behaviors. Eur. J. Psychol. 19, 158–173. doi: 10.5964/ejop.6559, 37731892 PMC10508211

[ref8] Cropanzano R. Mitchell M. S. (2005). Social exchange theory: an interdisciplinary review. J. Manage. 31, 874–900. doi: 10.1177/0149206305279602

[ref9] Cui Z. Zhang K. (2025). Is political skill always beneficial? The relationship between political skill and unethical pro-supervisor behavior. J. Manage. Organ. 31, 1737–1754. doi: 10.1017/jmo.2023.25

[ref10] DeFillippi R. J. Arthur M. B. (1994). The boundaryless career: a competency-based perspective. J. Organ. Behav. 15, 307–324. doi: 10.1002/job.4030150403

[ref11] Dulebohn J. H. Bommer W. H. Liden R. C. Brouer R. L. Ferris G. R. (2012). A meta-analysis of antecedents and consequences of leader-member exchange: integrating the past with an eye toward the future. J. Manage. 38, 1715–1759. doi: 10.1177/0149206311415280

[ref12] Epitropaki O. Kapoutsis I. Ellen B. P. Ferris G. R. Drivas K. Ntotsi A. (2016). Navigating uneven terrain: the roles of political skill and LMX differentiation in prediction of work relationship quality and work outcomes. J. Organ. Behav. 37, 1078–1103. doi: 10.1002/job.2100

[ref13] Erdogan B. Bauer T. N. (2014). “Leader-member exchange (LMX) theory: the relational approach to leadership,” in The Oxford Handbook of Leadership and Organizations, ed. Day D. V. (New York, NY: Oxford University Press).

[ref14] Ferris G. R. Anthony W. P. Kolodinsky R. W. Gilmore D. C. Harvey M. G. (2002). “Development of political skill,” in Research in Management Education and Development, Volume 1: Rethinking Management Education for the 21st Century, eds. Wankel C. DeFillippi R. (Greenwich, CT: Information Age Publishing).

[ref15] Ferris G. R. Davidson S. L. Perrewe P. L. (2005a). Political Skill at Work: Impact on Work Effectiveness. Palo Alto, CA: Davies-Black.

[ref16] Ferris G. R. Treadway D. C. Kolodinsky R. W. Hochwarter W. A. Kacmar C. J. Douglas C. . (2005b). Development and validation of the political skill inventory. J. Manage. 31, 126–152. doi: 10.1177/0149206304271386

[ref17] Ferris G. R. Treadway D. C. Perrewé P. L. Brouer R. L. Douglas C. Lux S. (2007). Political skill in organizations. J. Manage. 33, 290–320. doi: 10.1177/0149206307300813

[ref18] Frieder R. E. Ferris G. R. Perrewé P. L. Wihler A. Brooks C. D. (2024). The contingent nature of the political skill-employee performance relationship. J. Appl. Psychol. 109, 1132–1144. doi: 10.1037/apl0001107, 37307361

[ref19] Fugate M. Kinicki A. J. Ashforth B. E. (2004). Employability: a psycho-social construct, its dimensions, and applications. J. Vocat. Behav. 65, 14–38. doi: 10.1016/j.jvb.2003.10.005

[ref20] Gouldner A. W. (1960). The norm of reciprocity: a preliminary statement. Am. Sociol. Rev. 25, 161–178. doi: 10.2307/2092623

[ref21] Graen G. B. Uhl-Bien M. (1995). Relationship-based approach to leadership: development of leader-member exchange (LMX) theory of leadership over 25 years: applying a multi-level multi-domain perspective. Leader. Q. 6, 219–247. doi: 10.1016/1048-9843(95)90036-5

[ref22] Greenbaum R. L. Mawritz M. B. Bonner J. M. Webster B. D. Kim J. (2018). Supervisor expediency to employee expediency: the moderating role of leader-member exchange and the mediating role of employee unethical tolerance. J. Organ. Behav. 39, 525–541. doi: 10.1002/job.2258

[ref23] Han G. (2010). Trust and career satisfaction: the role of LMX. Career Dev. Int. 15, 437–458. doi: 10.1108/13620431011075321

[ref24] Hayes A. F. (2013). An Introduction to Mediation, Moderation, and Conditional process Analysis. New York, NY: Guilford Press.

[ref25] Hoobler J. M. Wayne S. J. Lemmon G. (2009). Bosses’ perceptions of family-work conflict and women’s promotability: glass ceiling effects. Acad. Manag. J. 52, 939–957. doi: 10.5465/AMJ.2009.44633700

[ref26] Jang E. (2022). Authentic leadership and task performance via psychological capital: the moderated mediation role of performance pressure. Front. Psychol. 13:722214. doi: 10.3389/fpsyg.2022.722214, 35712172 PMC9197480

[ref27] Janssen O. Van Yperen N. W. (2004). Employees’ goal orientations, the quality of leader-member exchange, and the outcomes of job performance and job satisfaction. Acad. Manag. J. 47, 368–384. doi: 10.2307/20159587

[ref28] Li C. J. Li K. Tu Y. Fan L. Xu C. Zhang H. (2025). Getting ahead in “murky waters”: political skill, organizational politics, and leader-rated employee promotability. Pers. Individ. Differ. 241:113188. doi: 10.1016/j.paid.2025.113188

[ref29] Magnusen M. Kim J. W. (2016). Thriving in the political sport arena: LMX as a mediator of the political skill-career success relationship. J. Appl. Sport Manag. 8, 15–42. doi: 10.18666/JASM-2016-V8-I3-6456

[ref30] Meeker B. F. (1971). Decisions and exchange. Am. Sociol. Rev. 36, 485–495. doi: 10.2307/2093088

[ref31] Mintzberg H. (1985). The organization as political arena. J. Manage. Stud. 22, 133–154. doi: 10.1111/j.1467-6486.1985.tb00069.x

[ref32] Muthén L. K. Muthén B. O. (2007). Mplus: Statistical Analysis with Latent Variables (Version 3) [Computer Software]. Angeles, CA: Muthén and Muthén.

[ref33] Organ D. W. (1988). Organizational Citizenship Behavior: The Good Soldier Syndrome. Lexington, MA: Lexington Books.

[ref34] Ployhart R. E. Vandenberg R. J. (2010). Longitudinal research: the theory, design, and analysis of change. J. Manage. 36, 94–120. doi: 10.1177/0149206309352110

[ref35] Podsakoff P. M. MacKenzie S. B. Lee J. Y. Podsakoff N. P. (2003). Common method biases in behavioral research: a critical review of the literature and recommended remedies. J. Appl. Psychol. 88, 879–903. doi: 10.1037/0021-9010.88.5.879, 14516251

[ref36] Raghuram S. Gajendran R. S. Liu X. Somaya D. (2017). Boundaryless LMX: examining LMX’S impact on external career outcomes and alumni goodwill. Pers. Psychol. 70, 399–428. doi: 10.1111/peps.12143

[ref37] Rao L. Chen Y. Li C. (2026). From frustration to skill: a moderated mediation model of how political frustration shapes political skill. Asia Pac. J. Manage., 1–36. doi: 10.1007/s10490-026-10119-8

[ref38] Rockstuhl T. Dulebohn J. H. Ang S. Shore L. M. (2012). Leader–member exchange (LMX) and culture: a meta-analysis of correlates of LMX across 23 countries. J. Appl. Psychol. 97, 1097–1130. doi: 10.1037/a0029978, 22985117

[ref39] Schaubroeck J. Lam S. S. K. (2002). How similarity to peers and supervisor influences organizational advancement in different cultures. Acad. Manag. J. 45, 1120–1136. doi: 10.2307/3069428

[ref40] Seibert S. E. Kraimer M. L. Liden R. (2001). A social capital theory of career success. Acad. Manag. J. 44, 219–237. doi: 10.2307/3069452

[ref41] Tang L. Yang F. Yang W. G. (2019). The effect mechanism of political skill on employee promotability. Econ. Manag. 41, 73–89. doi: 10.19616/j.cnki.bmj.2019.10.005

[ref42] Thacker R. A. Wayne S. J. (1995). An examination of the relationship between upward influence tactics and assessments of promotability. J. Manage. 21, 739–756. doi: 10.1016/0149-2063(95)90008-X

[ref43] Tiwari S. Jain V. Anis S. (2025). Variation of political skill dimensions across different industries. Vision J. Bus. Perspect. 29, 232–243. doi: 10.1177/09722629211065601

[ref44] Wang Y. Y. Duan J. Y. (2015). How does political skill influence employees’ voice: the roles of relationships and performance. Manage. World 3, 102–112. doi: 10.19744/j.cnki.11-1235/f.2015.03.010

[ref45] Wanigasekara S. K. Ali M. French E. (2026). Determinants of internal and external networking behaviours: insights into gender, education, political skills and mentoring. Hum. Resour. Dev. Int. 29, 269–295. doi: 10.1080/13678868.2024.2375952

[ref46] Wayne S. J. Shore L. M. Bommer W. H. (2002). The role of fair treatment and rewards in perceptions of organizational support and leader-member exchange. J. Appl. Psychol. 87, 590–598. doi: 10.1037/0021-9010.87.3.590, 12090617

[ref47] Wihler A. Blickle G. Iii B. P. E. Hochwarter W. A. Ferris G. R. (2017). Personal initiative and job performance evaluations. J. Manage. 43, 1388–1420. doi: 10.1177/0149206314552451

[ref48] Zahid F. Butt A. N. Malik M. A. R. (2025). When and how political skill becomes counterproductive: a moral licensing view. Aust. J. Manage. 50, 607–630. doi: 10.1177/03128962231205455

